# Exploring Narrative Ability in Greek-Speaking Children with High-Functioning ASD: Associations with Memory and Attention

**DOI:** 10.3390/brainsci15010073

**Published:** 2025-01-15

**Authors:** Vasiliki Zarokanellou, Alexandros Gryparis, Katerina Papanikolaou

**Affiliations:** 1 Department of Speech and Language Therapy, University of Ioannina, 45500 Ioannina, Greece; vzarokanellou@uoi.gr; 2 Department of Child Psychiatry, Agia Sophia Children’s Hospital, Medical School, National and Kapodistrian University of Athens, 11527 Athina, Greece; katpapan@med.uoa.gr

**Keywords:** narratives, narrative competence, microstructure, macrostructure, memory, ADHD

## Abstract

**Background/Objectives**: Narration is a sensitive tool for the assessment of language in children with high-functioning autism spectrum disorder (HF-ASD) since mild language deficits beyond the sentential level are not always noticeable through the administration of standardized language tests targeting the lexical or sentential level. This study investigated the narrative ability of monolingual Greek-speaking HF-ASD children in comparison to that of their typically developing (TD) peers and explored the associations between narrative variables, ADHD symptomatology, and memory skills in the participants on the autistic spectrum. **Methods**: The participants were 39 children aged 7 to 12 years, 19 with HF-ASD and 20 age-matched, vocabulary-matched, and cognitively matched TD peers. **Results**: The two groups were similar in most microstructural and macrostructural variables but differed significantly in syntactic complexity (*p* = 0.024; *d* = 0.754) and subordination (*p* < 0.001; *d* = −1.576) indices, implying that the HF-ASD group presented syntactic delay in comparison to their TD peers. The HF-ASD participants showed significantly higher heterogeneity in the amount of information generated for the story’s main character (*p* = 0.004; *d* = −0.093) in comparison to their TD peers. Significant associations were observed between verbal and visual memory, complex syntactic structures, and Theory of Mind-related internal state terms. ADHD symptomatology was negatively correlated with the generation of simple and coordinated clauses. Finally, complex syntax and delayed vSTM were correlated with retelling total scores, indicating that language ability and verbal memory compensate for narrative competence in HF-ASD children. **Conclusions**: The findings highlight the impact that language skills, memory ability, and ADHD symptomatology have on narrative competence in children with HF-ASD, as well as the importance of narrative use for assessing the language skills in populations with mild language impairment.

## 1. Introduction

Autism spectrum disorder (ASD) is a complex neurodevelopmental disorder that is associated with persistent impairment in social interaction and social communication across various environments, as well as restricted and repetitive patterns of behaviors, interests, and activities [1]. According to the Centers for Disease Control and Prevention [2], most children on the Autism spectrum present normal (41.7%) or borderline (23.1%) intellectual ability and are characterized as children with high-functioning ASD (HF-ASD). Even though these children have a normal range of intelligence, they commonly present significant language deficits. Findings from several studies propose high comorbidity between HF-ASD and Developmental Language Disorder (DLD) [3,4,5]. Children with ASD and normal intelligence form two distinct subgroups, one with language skills that fall in the normal range and one with concurrent DLD [5]. Relevant research suggests that children with HF-ASD present an uneven language profile with more profound language deficits in pragmatics, specifically in narrative skills, while language core abilities (syntax and semantics) can be relatively intact [6,7].

### 1.1. Narrative Skills in Children with HF-ASD

Narrative is described as a common type of discourse, involving the relation of a sequence of events [8]. The ability to narrate is significant for sharing experiences and communicating memories leading to the development of social and emotional relationships [9]. Narrative skills also play a major role in academic performance and lay the foundation for written production [9]. Narration is a complex task that incorporates language, cognitive, and social skills, starting to develop early, even before the age of 3, in typically developing (TD) children [10]. Research regarding narrative ability in children with HF-ASD reports inconsistent findings, which may be due to the wide age range of HF-ASD participants and the divergent matching criteria with the control groups used, as well as the different methodologies administered for the elicitation of narrations [8,9]. A meta-analysis including 24 studies reported significant mean differences in most microstructural and macrostructural variables measured in children with HF-ASD in comparison to their TD peers, but these findings presented moderate to high heterogeneity [9]. Regarding microstructure, several studies did not find significant differences in story length produced by children with HF-ASD and control groups [11,12,13,14,15,16], but some reported the opposite [7,9,17,18]. Studies examining the syntactic complexity of generated narratives suggest that the language ability of HF-ASD children significantly affects grammatical performance in narrative tasks [9,18]. Peristeri et al. [16] reported that HF-ASD children with language ability in the lower end of the normal range produced simpler and syntactically less complex sentences than their TD peers, while HF-ASD children with language ability in the upper end of the normal range performed syntactically similarly to the control group. In line with the above, several studies did not disclose significant differences in the mean length of utterances or other syntactic complexity measures between children with HF-ASD and their TD age-matched and/or language-matched peers [7,12,13,14,15,19], even though some did [17,18,20]. In contrast to the findings about microstructure, most studies referring to a macrostructure analysis (overall content of story and hierarchical sequencing of events) report significant difficulties in children with HF-ASD [9,11,17,18,21] in comparison to the TD language-matched and age-matched controls. These difficulties include a lower number of features described, fewer causal relations between events, more incoherent narratives containing more irrelevant comments, fewer inferences from the story events, and a less frequent reference to the cognitive states and emotions of the characters of the story [7,9,17].

### 1.2. Links Between Narrative Performance and Executive Functions in HF-ASD Children

“Executive functions” (EFs) include a broad range of purposeful upper cognitive processing skills serving for goal-directed behavior, abstract reasoning, decision-making, and social regulation [22,23]. A recent model of EFs divides them into a. cold (i.e., memory, inhibition, attention, cognitive flexibility, and monitoring), serving to manipulate abstract concepts, letters, and numbers, and b. hot, participating in emotional regulation, empathy, and the Theory of Mind (ToM) [24]. Deficits in EFs have a significant impact on generated narratives, and difficulties in narration in children with HF-ASD may be explained with reference to their cognitive differences [9]. HF-ASD children present important deficits in planning, cognitive flexibility, and attention, which play an instrumental role in the encoding of new information into episodes, the hierarchical organization of the episodes in a story, and the understanding of causal relationships between the narrated events [9]. Moreover, narration requires an intact working memory system since the speaker must remember what they narrated while planning the next utterance. Relevant research reveals that the narration of future episodes attests to supplementary working memory resources compared to the narration of past events from episodic memory, making the narration of future episodes a more difficult task [25]. Tsimpli et al. [26] suggest that higher performance in working memory (WM) is associated with better narrative competence in TD children. Along the same line, research in children with ASD shows that lower WM capacity is associated with lower syntactic complexity and less coherent and cohesive narrations [27,28,29]. Additionally, children with HF-ASD present a local over a global style of information processing, missing the gist of a story [12]. This deficit in central coherence makes the integration of new information into meaningful coherent events and the use of context to infer meaning more difficult, accounting for more superficial narratives with less causal relationships between events [7,8,9]. Finally, HF-ASD children have difficulties in empathizing; in understanding complex feelings such as jealousy, grief, regret, etc.; and in attributing mental states in themselves and others, and they often show a limited range of emotions [7]. These abilities are essential for the successful generation of a story because the speaker must “transmit” the feelings, thoughts, and conflicts of the main character of the story and not just describe the events. Moreover, the narrator has to take into consideration the reaction of the audience toward the story to adapt the narration accordingly [7,9]. Previous studies provide contradictory results with some proposing that children with HF-ASD produce fewer internal mental state terms (ISTs) [7], emotions [11], and causal explanations about the behavior of the main character of the story [11] than controls, while others did not report a difference between the two groups [10].

### 1.3. Purpose of This Study

The current study aims to investigate narrative competence in a group of monolingual Greek-speaking children with HF-ASD and to compare their narrative ability to that of a group of age-matched, vocabulary-matched, and cognitively matched TD peers. Furthermore, this study investigates the relationships between the microstructural and macrostructural indices on narrative tasks, ADHD symptomatology, and memory skills since previous research suggests that executive functions, specifically attention and memory skills, significantly affect narration in children with HF-ASD [27,28,29]. The research questions that the present study poses are the following:

1. Does the narrative microstructure (productivity and grammar) differ significantly in children with HF-ASD compared to their TD peers?
2. Does the narrative macrostructure differ significantly in children with HF-ASD compared to their TD peers?
3. Does ISL produced on narratives differ significantly in children with HF-ASD compared to their TD peers?

## 2. Materials and Methods

### 2.1. Participants

The participants included nineteen children with ASD and twenty typically developing (TD) peers, aged 7 to 12 years old. All the participants were monolingual Greek speakers and attended general elementary schools, each with a performance and verbal IQ over 70, as measured by Raven’s Educational [30]. The ASD children were recruited from a special ASD clinic in Athens and had taken a formal diagnosis of ASD without accompanying intellectual impairment (Level 1) according to the criteria of the Diagnostic and Statistical Manual of Mental Disorders (DSM)-5 [1] by an experienced child-psychiatrist specialized in ASD. The TD participants were recruited from different general elementary schools in two regions of Greece. They had free medical and educational history records and were typically developing according to their teacher and parent reports. The TD participants had scores below 11 on the Greek version of the Social Communication Questionnaire [31] and of 93% on the Greek Assessment Scale for ADHD-IV [32], as completed by their primary caregivers. The two groups were similar in terms of age, gender, IQ, naming ability, and verbal memory skills but presented significant differences in SCQ and ADHD total scores as expected (Table 1).

### 2.2. Materials and Measurements

#### 2.2.1. Screening Tools

1. *Raven’s Educational* [30]. Raven’s Educational is a screening norm-referenced instrument of general cognitive ability for monolingual Greek children aged 4;0 to 11;11 years old. It includes two separate scales, the Raven’s Colored Progressive Matrices (CPM), which assess performance intelligence (PIQ), and the Crichton Vocabulary Scales (CVS), which assess the production of definitions (verbal IQ). In the current study, it was employed as a criterion assessment instrument to ensure that all the participants had performance and verbal IQs over 70.
2. *Greek Assessment Scale for Attention Deficit Hyperactivity Disorder-IV* [32]. It is a screening standardized questionnaire of attention deficit hyperactivity disorder (ADHD) for children and adolescents aged 5 to 19 years old. The questionnaire has two forms, one for caregivers and one for the teacher of the class. In the current study, the caregiver’s form was used to exclude participants with possible ADHD in the control group and to assess ADHD symptomatology in the ASD group. For the parents’ form, a cut-off of 93% is recommended as the optimum point for possible ADHD with 74% sensitivity, 70% specificity, 72% positive predictive value, and 72% negative predictive value.
3. *The Greek version of the Social Communication Questionnaire (SCQ, Lifetime Form)* [31]. It is a screening instrument for ASD symptomatology, which is easily completed by the primary caregivers of the child in less than 10 min. It is appropriate for individuals over the age of 4 years old with cognitive functioning above the age of 2 and it investigates the full developmental history of each examinee. In this study, it was used to exclude participants with possible ASD in the control group and to assess ASD severity in the ASD group. An optimum cut-off of 15 points is recommended by the authors as positive for autism, while a score above 11 is recommended for HF-ASD in a Greek sample of 7–10-year-old children with high sensitivity and specificity (autism vs. TD children: 96.3% sensitivity and 98.7% specificity; HF-ASD vs. TD group: 90.6% sensitivity and 89.6% specificity).

#### 2.2.2. Naming Skills

*The Greek version of the Expressive One-Word Picture Vocabulary Test-Revised (EOWPVT-R)* [33]. It is a naming test of 100 words presented with colorful line drawings, evaluating expressive vocabulary in children aged 2 to 12 years old. In this task, the child must name the picture that the examiner shows. The target words comprise mainly high-frequency and low-frequency nouns and a few verbs, (e.g.,“watch” and “sew”). In this study, a Greek-translated and adapted version was used since in Greek there is no standardized naming test for children over the age of eight. The administration of the test always started from the beginning of the tool regardless of the age of the participant and terminated when the participant made six consecutive mistakes. The children were awarded one point for each correct answer, while no points were given for incorrect answers, leading to a possible maximum raw score of 100 points. The raw scores were used for between-group comparisons.

#### 2.2.3. Memory Skills

The Screening Scale of Visual and Verbal Memory from the Memory Assessment Battery for preschool and school-aged children [34] was implemented. It is a standardized instrument, which evaluates verbal and visual short-term and delayed short-term memory skills for children aged 5 to 12 years old.

For the assessment of verbal memory, participants have to recall a list of five or seven unrelated words depending on their chronological age once they hear them from the examiner. The words are produced by the examiner at a steady speaking rate of one word per second. Each examinee has five trials to succeed in this goal. Every correctly recalled word gives one point, and once the examinee has recalled all the words accurately in one attempt, they get five points for all remaining trials. Afterward, independently of the number of correctly recalled words from each examinee, the examiner asks the participant once again to recall the list of words, giving a distinctive cue for each of them, e.g., for the word trousers, the examiner says the following to the child: “We wear it”. This is done to ensure that participants have adequately encoded the information. After a break of 5 to 10 min, the examiner asks the examinee to reproduce the same list of words once again.

For the assessment of visual memory, participants have to recall the positions of five or seven monochrome tokens, depending on their chronological age, once they have seen the positions they are placed in by the examiner on a white table board with squares. Similar to the verbal memory task, each examinee has five trials to succeed in this goal. Every correctly recalled position οf a token gets one point, and once the examinee has recalled all the positions of the tokens correctly in one attempt, they get five points for all remaining trials. After a short interval of 5 to 10 min, where another verbal task can be administered, the examiner asks the examinee to reproduce the same positions of the tokens once again.

#### 2.2.4. Narrative Ability

The participants’ oral retellings were elicited by using the two short stories of the “Storytelling Scale” from the Memory Assessment Battery for preschool and school-aged children [34]. The stories were presented orally and without visual support for retelling, according to the manual of the test. These stories were chosen as they are culturally and socially neutral. Furthermore, their themes are carefully selected to be familiar to the children, triggering their interest, and they are also compatible with the age of the participants. For each story, to assess the microstructure, the following linguistic indicators were measured as proposed by the relevant literature [9,16]:

(1) Total number of words: The total number of words produced in the two retelling tasks was compared between the children with HF-ASD and their TD peers.
(2) Lexical diversity: The lexical diversity index is calculated as the number of different content words divided by the total number of content words. The lexical diversity indices were compared between the two study groups.
(3) Syntactic complexity: The syntactic complexity index is measured as the number of complex sentences, which include subordinate and coordinate clauses, divided by the total number of simple and complex clauses.
(4) Subordination index: It is calculated as the number of subordinate clauses divided by the total number of complex sentences
(5) Types of subordination: According to Peristeri et al.’s [16] study, the types of subordination were investigated, including the total number of verb-complement clauses and modifiers, such as adverbial and relative clauses. Ιn complement clauses, the type of complementizers (subjective na vs. indicative oti/pos and factive pu vs. non-factive oti/pos) were further examined. In Greek, the complementizer na is the least complex and the first to be acquired by native Greek-speaking children [16].

For the assessment of the macrostructure, the following information units were evaluated, as the relevant literature proposes [9,16]: a. the information given about the place and the time that the story occurred, b. the information given about the main characters of the story, c. the information given about the event content of the story, d. the information given about the conflict and the attempts to resolve the main problem, and e. the information given about the final result and the conclusions of the story. Each component of macro narrative skills was assessed with 0, 1, 2, or 3 points, giving a total possible score of 15 points for each narrative task. Moreover, the following were calculated: the total number of +ToM-related ISTs (emotion and mental verb ISTs) and the total number of −ToM-related ISTs, including the number of perceptual, physiological, and linguistic action verbs [7,16].

Finally, the transcription of each narrative was made by this study’s first author, an SLP with more than 15 years of clinical experience. An independent rater with a background in linguistics analyzed all the transcripts for grammatical microstructure variables and ±ToM ISTs. Subsequently, the first author of this study re-evaluated all the transcripts for microstructure and macrostructure variables. Both raters were blind to the status of the child that produced each narrative. Disagreements between the two raters were resolved through discussion. Spearman’s correlation coefficients between the first and the second rater were excellent. Specifically, Spearman’s correlation coefficients for attested grammatical microstructure variables exceeded 0.95 (*p* < 0.001), while for ±ToM ISTs, they exceeded 0.97 (+ToM ISTs: *rho* = 0.971 and *p* < 0.001; −ToM ISTs: *rho* = 0.984 and *p* < 0.001).

### 2.3. Procedure

**Figure 1:**
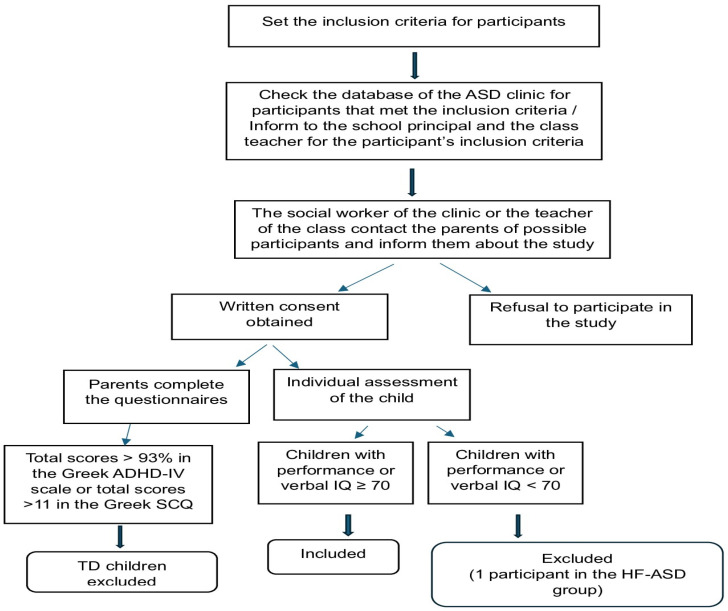
Procedure flow diagram.

Before the beginning of this study, approval was received by the Ethics Committee of the Greek Institution of Educational Policy (Φ15/176437/204757/Δ1) and the Ethics Committee of the Medical School. According to the research protocol, all the caregivers provided a written consent form and completed the Greek version of the SCQ, Lifetime Form [31], as well as the Greek Assessment Scale for Attention Deficit Hyperactivity Disorder-IV [31], before the individual assessment of each child. For the cognitive evaluation of the participants, the Greek edition of Raven’s Educational [30] was administered, followed by the Screening Scale of Visual and Verbal Memory [34]. Lastly, the EOWPVT-R [33] was given followed by the two narrative tasks. The tests were administered in the same order to all the participants. Each participant was evaluated individually in a quiet room free of distraction. The participants with ASD were assessed in the clinic, from where they were recruited, while their TD peers were assessed in either their home environment or in their school setting. All the answers of the participants were audio-recorded and then transferred to their corresponding answer sheets. Overall, the procedure followed for the assessment of the participants is pictured in the following diagram (Figure 1):

### 2.4. Statistical Analysis

The quantitative variables were expressed as the mean and standard deviation. The qualitative variables were expressed as absolute and relative (%) frequencies. For the comparison of proportions, the chi-square test was used. Student’s *t*-test was used for the comparison of the continuous variables between two groups. Spearman’s correlation coefficient was used to investigate the linear relationship between the micro- and macrostructure indices and cognitive variables in the ASD group. The effect sizes were calculated using Cohen’s d coefficient. Effect sizes up to 0.2 are considered small, between 0.2 and 0.5 are moderate, sizes between 0.5 and 0.8 are considered large, and sizes over 0.8 are considered very large [35]. Finally, the inter-rater reliability of the narrative measures between two independent raters was calculated using Spearman’s correlation coefficient. All the reported *p*-values are two-tailed. Statistical significance was set at *p* < 0.05 and the analyses were conducted using IBM SPSS v29 [36].

## 3. Results

### 3.1. Microstructure Measures

The two groups were similar in terms of vocabulary productivity since they performed alike in the lexical diversity index (*p* = 0.079; *d* = −0.002) and in the total number of words (*p* = 0.976; *d* = −0.0383) they generated during the narration of the two stories (Table 2). Also, the two groups used an equivalent number of coordinate (*p* = 0.563; *d* = 0.504), subordinate (*p* = 0.554; *d* = −0.050), verb-complement (*p* = 0.887; *d* = 0.064), and modifier clauses (*p* = 0.775; *d* = 0.291) in their narrations (Table 2). However, the TD peers produced significantly more simple clauses than the ASD participants (*p* < 0.001; *d* = 2.205) and they used more coordinate clauses in their retellings than the ASD children (*p* = 0.563; *d* = 0.504) even though this difference did not reach statistical significance. Finally, the TD participants showed higher syntactic complexity (*p* = 0.024; *d* = 0.754) and subordination (*p* < 0.001; *d* = −1.576) indices in relationship to their ASD peers, indicating that the ASD group presented syntactic delay in comparison to the TD group. The above findings are presented in the following table (Table 2).

### 3.2. Macrostructure Measure

The two groups performed similarly in all the macrostructure measures except the information describing the story’s main character (*p* = 0.004; *d* = −0.093), in which the participants with ASD presented significantly higher heterogeneity in the amount of information generated in comparison to their TD peers (TD: mean = 4.8 and *SD* = 0.6 vs. ASD: mean = 4.8 and *SD* = 1.3). The results are presented in Table 3.

### 3.3. Correlations Between Microstructure and Macrostructure Measures in the ASD Group

To investigate the extent to which the core language abilities (syntax and vocabulary) impact retelling skills in the ASD group, we examined the correlation between the microstructural and macrostructural measures. The statistical analysis revealed significant correlations between the coordinate clauses and story total scores (*rho* = 0.5; *p* < 0.05), subordinate clauses and story total scores (*rho* = 0.5; *p* ≤ 0.01), and complex clauses and story total scores (*rho* = 0.6; *p* ≤ 0.01). Finally, the statistical analysis showed significant correlations between the +ToM ISTs and subordinate clauses (*rho* = 0.4; *p* < 0.05), +ToM ISTs and complex clauses (*rho* = 0.4; *p* ≤ 0.01), −ToM ISTs and subordinate clauses (*rho* = 0.6; *p* ≤ 0.01), and −ToM ISTs and complex clauses (*rho* = 0.5; *p* ≤ 0.01). The results are presented in Table 4.

### 3.4. Correlations Between Language and Executive Function Measures in the ASD Group

To begin with, ADHD symptomatology was associated significantly and negatively with the generation of simple (*rho* = −0.3; *p* < 0.05) and coordinate (*rho* = −0.3; *p* < 0.05) clauses. As it concerns the syntactic complexity index, it was not related to any executive function measure. However, the generation of complex clauses was associated with immediate verbal (*rho* = 0.4; *p* < 0.05) and visual (*rho* = 0.4; *p* < 0.05) memory skills, as well as delayed verbal (*rho* = 0.4; *p* ≤ 0.01) and visual (*rho* = 0.5; *p* ≤ 0.01) memory skills. Similarly, subordinate clauses presented significant associations with all the memory variables. Finally, modifier clauses were associated with all the memory tasks, except delayed visual memory, while verb-complement clauses were related only to vSTM ability (*rho* = 0.3; *p* < 0.05). Regarding the macrostructure language measures, the retelling total scores were associated only with delayed vSTM (*rho* = 0.3; *p* < 0.05), while the production of emotion words and mental state verbs (+ToM ISTs) was related to all the memory tasks. Lastly, the generation of perceptual, physiological, and linguistic action verbs (−ToM ISTs) was associated only with vSTM ability (*rho* = 0.3; *p* < 0.05). The correlational analysis is presented in Table 5.

## 4. Discussion

The present study a. compared the syntactic and story structure complexity of narratives of monolingual Greek-speaking children with HF-ASD and neurotypical controls matched in age, gender, and cognitive abilities (IQ scores and memory skills), expanding on findings by previous research in Greek [15,16,19], and b. investigated the associations between the linguistic indicators of narratives, ADHD symptomatology, and memory abilities in a Greek-speaking sample. Previous research in ASD children supports that narrative competence correlates with language ability [9,16]. Additionally, the existing literature suggests that executive deficits, specifically deficits in attention and memory ability, significantly impact the narrative competence of children on the autistic spectrum [26,27,28].

Regarding the first question of this study, if children with HF-ASD differ significantly in comparison to their TD peers in narrative microstructure (productivity and grammar), the analysis revealed that the HF-ASD participants had similar performance to their TD peers in most microstructure variables. Analytically, the ASD group did not differ from the control group in story length and lexical diversity, reinforcing similar outcomes from other research in English and Greek [11,12,13,14,15,16].

Furthermore, the ASD group performed similarly in the production of coordinate and subordinate clauses compared to the control group but presented a significantly lower subordination index in comparison to their TD peers, a finding attributed to the use of more complex clauses by the control group. Moreover, the TD group had higher performance in coordinate sentences than the HF-ASD group and differed also significantly in the syntactic complexity index in relationship to the control group, supporting previous findings in Greek that claim that ASD-HL children produce less complex morpho-syntactic structures than their TD peers [15,20]. Additionally, Peristeri et al. [16] propose that the frequency of the use of coordinate clauses is related to language ability in ASD. Thus, the findings of our analysis align with the above suggestions, indicating that our HF-ASD group presented syntactic delay compared to their neurotypical peers [15,16,20]. A more detailed analysis of the complementizers used by the two groups of participants revealed that the ASD group did not differ from the control group in any variable. These findings contradict the results of Peristeri et al.’s [16] study, in which the ASD-HL group produced significantly fewer modifier clauses compared to the control group. This difference may be attributed to the different narration tasks used in the two studies. In the Edmonton Narrative Norms Instrument (ENNI) [37] used by Peristeri et al. [16], the child first hears a story and then must generate it by looking at its pictures, while in the present study, a retelling story task without pictures was used. Moreover, in Peristeri et al.’s [16] study, modifiers were significantly correlated with overall story structure complexity, a finding also not observed in this study. Previous research has proved that story materials influence the semantic quality and syntactic structures of the narratives generated in TD children [25] and children with ASD [11,38].

Regarding the performance of the two groups on macrostructure variables, the two groups performed alike, except in the amount of information generated about the characters of the story, where the HF-ASD group showed significantly greater variability in the number of features (family relationships and/or causal explanations of the behavior) described but not in the number of terms used to describe the main character’s cognitive states and emotions. The above aligns with previous results [15,38,39] and corroborates the Losh and Capps [11] findings, in which ASD participants provided fewer causal explanations about the behavior of the main character of the story than controls. Finally, there was a significant association between the story’s total scores and coordinate, subordinate, and complex clauses, revealing that language skills correlate with narrative competence, a result enhancing the outcomes from other studies in Greek [16].

Lastly, as to the third question of this study, the qualitative analysis of the narratives showed that both groups produced an equivalent number of ±ToM ISTs. These results contradict the findings of Peristeri et al. [16], in which the ASD-HL group produced significantly fewer −ToM ISTs, but are in line with the findings of Baldimitsi et al. [15], according to which inferior performance was not detected between the monolingual and bilingual HF-ASD children and their controls in the number of generated ±ToM ISTs. On the contrary, in the Baldimitsi et al. [15] study, the monolingual HF-ASD children generated significantly more −ToM ISTs compared to their monolingual controls. Finally, all the ±ToM ISTs were significantly correlated with subordinate and complex clauses since the use of these terms requires the generation of specific complementizers in Greek, such as oti/pu and na [16].

In addition to the above, significant associations between both verbal and visual memory skills and microstructure and macrostructure variables in the ASD group were observed. Also, a significant negative association between simple and coordinate clauses, and total scores of ADHD symptomatology, was affirmed. Retelling a story requires that the narrator can regenerate the conceptual representation of the story [39]. The production of the macrostructure of a story relies on global processing skills (central coherence and ToM abilities), which play a pivotal role in the understanding of the causal relationships between the different events and in the interpretation of the thoughts and feelings of characters [9,39], as well as on cognitive mechanisms, such as attention and memory [28]. An adequate attention span and good short-term memory skills are required for encoding the new information into episodes, while an intact working memory system is needed to actively hold what the speaker narrated while planning the next utterance [28]. In the current study, significant associations were established between delayed vSTM and retelling total scores, as well as between +ToM ISTs and vSTM and delayed vSTM, a result that supports the bilateral relationship between retelling story ability and memory skills, and ToM skills and working memory abilities. Moreover, there was a significant association between +ToM ISTs and immediate and delayed visual memory, but a similar association between −ToM ISTs and visual memory was not revealed. The above may be accredited to the different psycholinguistic features of the two categories of ±ToM ISTs. Possibly, the +ToM ISTs (emotions and mental verbs) are words presenting higher imageability than −ToM ISTs, and for their acquisition, individuals may activate and rely more on visual memory.

As concerns the associations between microstructure variables and memory skills, previous studies on TD children and children with ASD show a robust link between verbal working memory skills and syntactic capacity [29]. Specifically, working memory seems to get involved more heavily in the processing of complex sentences and not in simple sentences that contain fewer syntactic operations [29]. According to the regeneration of syntax hypothesis, the immediate recall of a sentence is not based on a surface representation of a string of words in memory but is reproduced by a conceptual representation stored in the long-term memory involving recently activated words and a normal mechanism of sentence production, thus enabling the verb of the sentence to determine the syntactic structure of the to-be-recalled sentence [40,41]. In the current study, the statistical analysis revealed significant associations between complex and subordinate clauses and immediate and delayed verbal and visual STM, giving evidence to previous findings, which claim that there is a strong association between verbal memory capacity and syntax in children with ASD [29]. Moreover, the modifier clauses were significantly correlated with immediate and delayed vSTM, as well as immediate visual memory, while verb-complement clauses were only associated with vSTM. A possible explanation for this disparity may relate to the information that carries each type of clause. Adverbial clauses provide temporal or causal information that modifies the event of the main clause [16] and for that reason may correlate also with visuospatial memory. Finally, ADHD symptomatology was negatively associated with simple and coordinate clauses. Another study has found significant associations between inattention and pragmatic skills in children with ASD and our findings are in line with these results [28].

## 5. Conclusions

Overall, the results of the present study highlight the important role of attention and working memory ability in the regeneration of story retellings in children with HF-ASD. The scarce research that has investigated the link between working memory skills and syntax has found a strong association between working memory capacity and the processing of complex syntactic structures and the current study affirms this link. Also, the results of this study confirm previous findings in Greek attesting to a similar pattern of macrostructure and microstructure narrative performance for children with HF-ASD to that of their neurotypical peers [15,16]. A limitation of the current study is the small sample of participants, which may have limited the statistical power of our findings. As a result, caution should be exercised when interpreting the findings, and further studies with larger sample sizes are warranted to confirm these observations. Another limitation is the non-implementation of a standardized narration task that would permit the direct comparison of the narrative performance of HF-ASD children to the mean performance of a larger sample of their neurotypical peers. Moreover, the language level of the participants was not evaluated with a standardized language tool such as CELF-4 [42], nor were the two study groups matched on standardized language scores. This would have permitted a more detailed examination of the relationship between language ability and narrative competence. In Greek, however, there is not a norm-referenced language test for children older than seven years old, nor is there a standardized assessment tool for narration. Even though this fact has limited the exploration of the associations between language skills and narrative ability, it also underscores the importance of using narratives as a sensitive tool for the assessment of language in children with HF-ASD since mild language deficits beyond the sentential level may not always be notable through the administration of standardized language tests targeting the sentential or lexical level. Future studies should examine to a greater extent the association of narrative indices to executive functions such as working memory and attention in HF-ASD children.

## Figures and Tables

**Table 1:** Characteristics of participants.

	Group			
	TD (*n*1 = 20)	ASD (*n*2 = 19)		
	Mean (SD)	Mean (SD)	*p*	*Cohen’s d*
Age (months)	118.2 (17.7)	118.0 (17.6)	0.946	0.014
Gender, Ν (%)				
Boys	15 (75%)	15 (78.9%)	0.770	
Girls	5 (25%)	4 (21.1%)		
Total score SCQ	5.2 (2.5)	13.0 (7.4)	0.002	−1.439
Total score ADHD	8.4 (5.1)	22.3 (12.5)	<0.001	−1.468
EOWPVT-R (RS)	69.9 (9.6)	68.4 (8.9)	0.864	0.160
Raven’s Educational				
Performance IQ	108.8 (15.8)	109.2 (14.5)	0.764	−0.030
Verbal IQ	96.5 (12.2)	89.0 (12.4)	0.864	0.615
Memory Assessment Battery				
Verbal short-term memory (RS)	27.3 (4.6)	28.4 (6.0)	0.919	−0.201
Delayed verbal short-term memory (RS)	6.1 (1.1)	5.8 (1.9)	0.076	0.169
Immediate visual memory (RS)	31.6 (4.4)	30.5 (5.6)	0.091	0.215
Delayed visual memory (RS)	6.4 (0.9)	5.7 (2.0)	0.012	0.473

**Table 2:** Group means (and SDs) of total scores for microstructure measures.

Total Scores of Microstructural Measures	TD Group *(n*1 = 20)	ASD Group (*n*2 = 19)	*p*-Value	Effect Size *Cohen’s d*
Total number of words	139.5 (31.6)	151.6 (31.9)	0.976	−0.383
Lexical diversity (%)	0.8 (0.04)	0.8 (0.05)	0.079	−0.002
Simple clauses	12.6 (3.1)	5.7 (3.1)	<0.001	2.205
Coordinate clauses	7.1 (2.7)	5.8 (2.2)	0.563	0.504
Subordinate clauses	8.7 (4.1)	8.9 (3.6)	0.554	−0.050
Syntactic complexity (%)	0.7 (0.2)	0.6 (0.1)	0.024	0.754
Subordination index (%)	0.6 (0.1)	0.4 (0.1)	<0.001	−1.576
Na complementizer	3.4 (1.7)	3.6 (1.8)	0.578	−0.102
Oti/pu complementizers	0.9 (1.0)	0.5 (0.7)	0.110	0.364
Verb-complement clauses	4.3 (2.2)	4.2 (2.3)	0.887	0.064
Modifier clauses	4.9 (2.0)	4.3 (2.0)	0.775	0.291

**Table 3:** Group means (and SDs) of total scores for macrostructure measures.

Total Raw Scores of Macrostructural Measures	TD Group (*n*1 = 20)	ASD Group (*n*2 = 19)	*p*-Value	Effect Size *Cohen’s d*
Place/Time	2.5 (1.7)	2.5 (1.7)	0.874	−0.014
Character/s	4.8 (0.6)	4.8 (1.3)	0.004	−0.093
Event Structure	4.3 (2.0)	3.2 (2.3)	0.230	0.487
Conflict–Solution	3.8 (1.2)	3.6 (1.2)	0.663	0.145
Conclusion	4.8 (1.1)	4.4 (1.5)	0.111	0.249
Total Scores	20.0 (4.2)	18.6 (5.1)	0.271	0.304
**Internal State Language**				
+ToM ISTs	7.0 (3.1)	6.6 (2.9)	0.438	0.140
−ToM ISTs	6.8 (2.7)	7.9 (2.8)	0.752	−0.400

**Table 4:** Spearman’s correlation coefficients between the microstructural and macrostructural variables in the ASD group.

Macrostructure Variables	Microstructure Variables						
	Syntactic Complexity	Simple Clauses	Coordinate Clauses	Subordinate Clauses	Complex Clauses	Verb-Complement	Modifier
+ToM ISTs	0.1	0.2	0.3	0.4 *	0.4 **	0.1	0.2
−ToM ISTs	−0.3	−0.0	0.3	0.6 **	0.5 **	−0.1	−0.1
Story total scores	0.2	0.0	0.5 **	0.5 **	0.6 **	0.1	−0.0

* *p* < 0.05; ** *p* ≤ 0.01.

**Table 5:** Spearman’s correlations between cognitive, microstructural, and macrostructural variables in the ASD group.

Variables	RS ADHD	vSTM	Delayed vSTM	Immediate Visual Memory	Delayed Visual Memory
Microstructure					
Syntactic complexity	−0.2	0.0	0.0	0.1	−0.0
Simple clauses	−0.3 *	−0.0	0.0	0.2	0.3
Complex clauses	−0.3	0.4 *	0.4 **	0.4 *	0.5 **
Coordinate clauses	−0.3 *	0.0	0.1	0.2	0.4 *
Subordinate clauses	−0.1	0.4 *	0.3 *	0.4 **	0.4 **
Verb-complement	0.2	0.3 *	0.3	0.3	0.1
Modifier	−0.0	0.3 *	0.4 **	0.4 **	0.3
Macrostructure					
Retelling total scores	−0.1	0.3	0.3 *	0.2	0.1
+ToM IST	0.1	0.4 **	0.6 **	0.5 **	0.6 **
−ToM IST	0.1	0.3 *	0.2	0.2	0.2

* *p* < 0.05; ** *p* ≤ 0.01.

## Data Availability

The data that support the findings of this study are available on request from the corresponding author. The data are not publicly available due to privacy or ethical restrictions.
